# Persistence of maternal antibodies against goat pox virus in goat kids

**DOI:** 10.1111/jvim.17135

**Published:** 2024-07-31

**Authors:** Mostafa Abdollahi, Mohsen Lotfi, Samad Lotfollahzadeh, Mohammad Reza Mokhber Dezfouli, Maryam Adibi, Morteza Kamalzadeh, Sajjad Firuzyar

**Affiliations:** ^1^ Department of Clinical Sciences, Faculty of Veterinary Medicine Semnan University Semnan Iran; ^2^ Department of Quality Control Razi Vaccine and Serum Research, Education and Extension Organization (AREEO) Karaj Iran; ^3^ Department of Internal Medicine, Faculty of Veterinary Medicine University of Tehran Tehran Iran

**Keywords:** goat kid, goat pox, passive immunity, vaccine

## Abstract

**Background:**

In goat kids, choosing the appropriate age to administer the first dose of goat pox disease (GTP) vaccine requires knowing when maternal antibody decline concentrations.

**Objective:**

Determine the persistence of maternal antibodies against goat pox virus (GTPV) in goat kids.

**Animals:**

Twenty Saanen goat kids from birth to 120 days old.

**Methods:**

In 2 groups, including: control (receiving colostrum from nonvaccinated does) and treatment (receiving colostrum from vaccinated does). On zero, 3, 7, 14, 21, 28, 42, 56, 70, 100 and 120 days after the birth, virus neutralization test was used to measure the serum concentration of antibodies against GTPV.

**Results:**

At the age of 56 days, the first seronegative goat kids (n = 2) were recorded in the treatment group. At the age of 120 days, all the goat kids in the treatment group were seronegative. The average virus neutralization index (VNI) of the goat kids became negative at the age of 100 to 120 days. All goat kids in the control group were negative at all times.

**Conclusions and Clinical Importance:**

One hundred to 120 days of the age seems to be the time to administer the first GTP vaccine in the goat kids with passive immunity against goat pox.

**Abbreviations**
FPT: failure of passive transfer
GTP: goat pox disease
GTPV: goat pox virus
VN: virus neutralization
VNI: virus neutralization index

## 1 INTRODUCTION

Goat pox (GTP) is of critical importance to goat production in multiple regions of the world (Middle East, Asia, North Equatorial Africa, and some parts of Europe) with severe economic consequences.^1^ GTP is an acute infectious disease characterized by fever, skin and internal pox lesions, rhinitis, conjunctivitis, enlarged lymph nodes and secondary pneumonia. The severity of disease is greater in young animals.^2^ GTP is caused by goat pox virus (GTPV), which belongs to the genus Capripox and the family Poxviridae.^3^, ^4^, ^5^ Although GTP outbreaks are reported in all months of the year, they often occur between November and May, with outbreaks peaking during March.^6^ In some areas, such as Iran, the seasonal pattern of elevated GTP incidence coincides with the period of peak goat kid population.^6^, ^7^ This justifies the importance of early GTP vaccination in goat kids.

Vaccination is the cheapest and the most sustainable means of controlling GTP in endemic countries.^8^ In Iran, annual vaccination with Gorgan strain GTPV attenuated live vaccine (Razi Institute, Karaj, Iran) is used to control GTP in goat herds. The timing of this vaccination in most flocks is before breeding.

Passive immunity acquired by maternal antibodies in colostrum is important in the health of a newborn.^9^ However, response to vaccination requires timing of administration after the decline of passive immunity of the newborn.

The occurrence of a good endogenous humoral immune response to vaccination requires reduction of the passive immunity of the newborn.^10^, ^11^ A comprehensive study on the persistence of maternal antibodies against GTPV in goat kids has not been done. The aim of this study is to determine the persistence of maternal antibodies against GTPV in goat kids that received adequate colostrum from the vaccinated does.

## 2 MATERIALS AND METHODS

### 2.1 Study area

This study was performed in a closed farm with 500 Saanen goats (located in Tehran, Iran) during the period from August 2022 to August 2023. The sample size was determined based on previous studies.^12^, ^13^, ^14^

### 2.2 Animals

**FIGURE 1:**
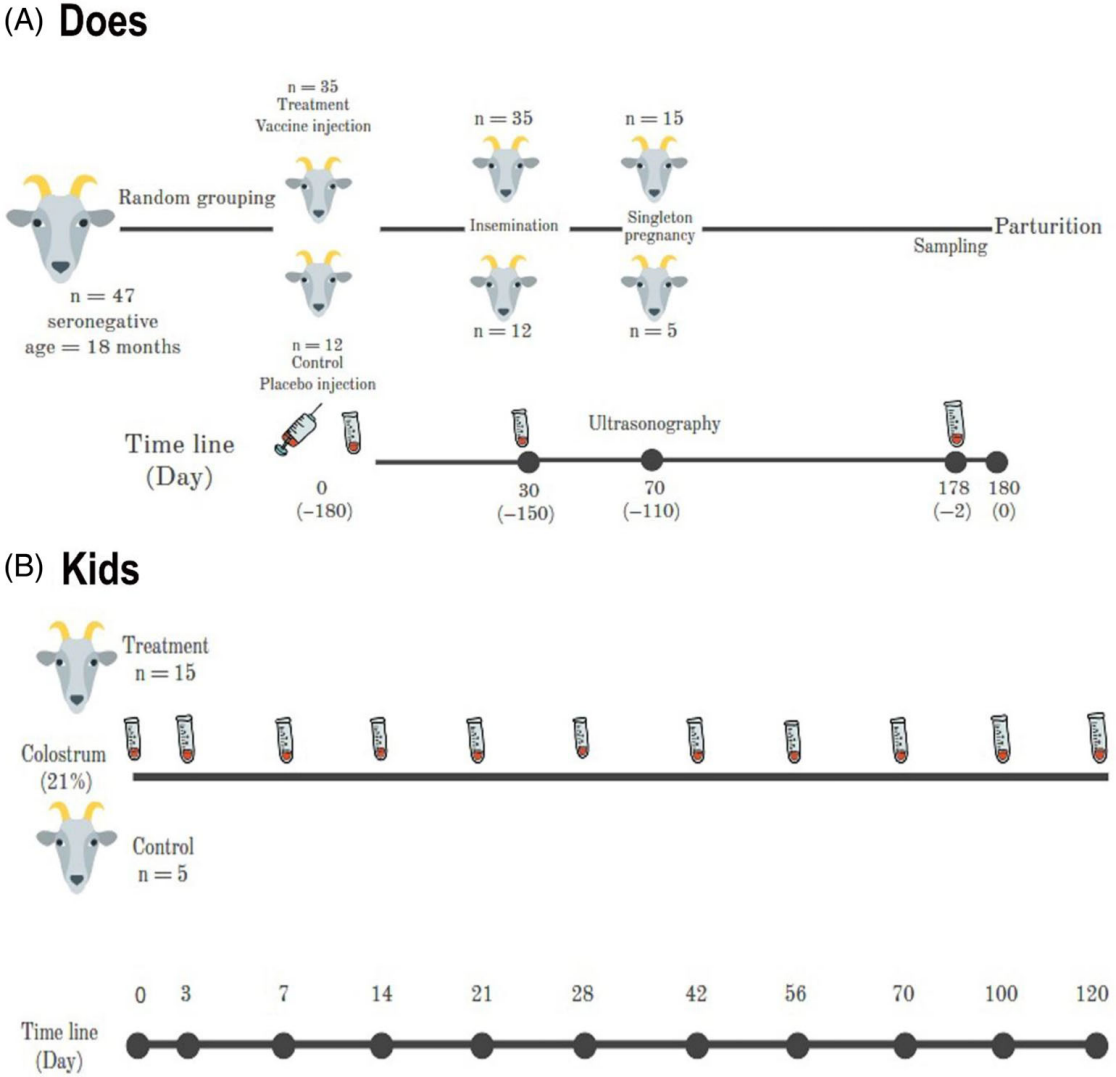
Study design in does and kids participating in the study.

Does: The studied animals included 47 seronegative 18 months old Saanen female goats. These animals were randomly divided into treatment (n = 35, receiving Gorgan strain attenuated live GTP vaccine) and control (n = 12, receiving sodium chloride 0.9% as placebo) groups.^15^ Forty days after transcervical artificial insemination, all does were examined via ultrasonography.^16^ Only goats with a singleton pregnancy remained in the study, including 15 goats in the treatment group and 5 goats in the control group (Figure 1).

Goat kids: All kids born by the studied does had appropriate birth weight (2.5‐3.5 kg) and were included in the study. After birth, kids were fed colostrum (200 mL immediately after birth and 210 mL/kg in the first 24 hours).^17^ Kids born by the does receiving the vaccine were in the treatment group (*n* = 15 including 8 males and 7 females) and kids born by the does receiving the placebo (*n* = 5 including 3 males and 2 females) were in the control group. Measurement of IgG serum concentration by ELISA method (DEIA640, CD Creative Diagnostics, Shirley, USA) at 48 hours after birth showed that all the studied kids had no failure of passive transfer (FPT) [cut‐off: 10 mg/mL].

### 2.3 Entry and exit criteria

Inclusion criteria determined for does (complete clinical health, singleton pregnancy, having a good Body Condition Scoring [3 on the 5‐point scale]) and for kids (complete clinical health, delivery without intervention, appropriate weight).^13^, ^18^

Exclusion criteria determined for does (disease incidence) and for kids (FPT, disease incidence).^14^, ^18^

### 2.4 Vaccination

The national protocol of Iran for vaccination against GTP is to prescribe 1 dose of live attenuated vaccine per year (annual vaccination). The administration of the mentioned vaccine is usually done before the breeding season. The study was designed to consider conditions according to industry standards. Vaccine and placebo were administered 30 days via subcutaneous injection into the front of the shoulder before artificial insemination. For vaccination, live attenuated Gorgan strain GTP vaccine was used. Each dose of the vaccine contained 10^5.7^ TCID_50_/mL of virus.

### 2.5 Serum collection

In does, blood samples were taken on the vaccination day and repeated 30 days after vaccination and 2 days before parturition (day 148 of pregnancy; Figure 1). In newborn kids, the first blood sample was taken immediately after the birth (before receiving colostrum), and then, on days 3, 7, 14, 21, 28, 42, 56, 70, 100 and 120 after birth (Figure 1).

### 2.6 Serological analysis

Virus neutralization (VN) test was used to determine the serum level of goat pox antibody. This test was performed according to the Office International des Epizooties (OIE) instructions.^12^, ^19^, ^20^ Briefly, serum samples were diluted with DMEM at 1 : 5 ratio and were inactivated by heating at 56°C for 30 minutes. Next, 50 μL of each serum sample was added to the wells of 2 columns of the microplate. Then, the GTPV (10^5.7^ TCID_50_/mL) was diluted in a log dilution series of log10^5.0; 4.0; 3.5; 3.0; 2.5; 2.0; 1.5^ TCID_50_ per milliliter. Starting with row G and the most diluted virus preparation, 50 μL of the virus was added to each well in that row. This step was repeated with each virus dilution so that the highest titer virus dilution was placed in row A. The microplate was incubated at 37°C for 1 hour. Then, a 100 μL of Lamb kidney cell suspension (10 000 cells per well) was added to each well, and the microplate was reincubated at 37°C and 5% CO_2_ atmosphere, for 9 days. During incubation, the microplates were examined daily for cytopathic effect, and the serum titer was calculated by the Karber method. The difference between the test serum titer and the negative serum titer was calculated as the virus neutralizing index (VNI). According to the OIE instructions (OIE, 2017), VNI ≥1.50 was considered as positive.

### 2.7 Statistical analysis

The normal distribution of data was confirmed using Shapiro‐Wilk test. After plotting the serum VNI‐time diagram in GraphPad Prism software (Version 9), independent *t*‐test in SPSS software (Version 22) was used for statistical analysis of data. Statistically, significant differences were declared at a *P* value of less than .05.

## 3 RESULTS

**FIGURE 2:**
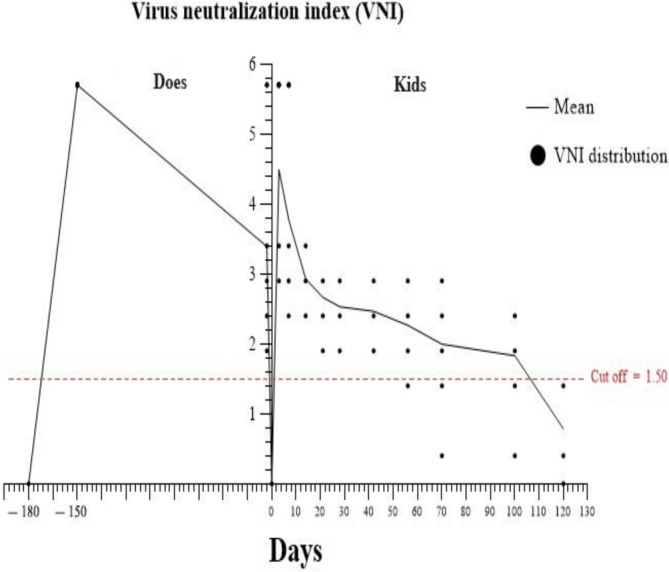
VNI‐time diagram in does and kids participating in the study.

Does: As shown in Table 1 and Figure 2, on 180 days before parturition, there were no significant differences in VNI between the control and treatment groups (*P* > .05). On 150 and 2 days before parturition, VNI was significantly higher in the treatment group as compared with VNI in the control group (*P* < .05).

**TABLE 1:** Virus neutralization index (VNI) in does and kids participating in the study (mean ± SD).

	Treatment group			Control group			
Animals	Day	VNI	VN ability	VNI	VN ability	*P* value	Interpretation
Does	−180	0.0 ± 0.0	−	0.0 ± 0.0	−	1.000	*μ*_*T*_ = *μ*_*C*_
Does	−150	5.7 ± 0.0	+	0.0 ± 0.0	−	.001	*μ*_*T*_ > *μ*_*C*_
Does	−2	3.4 ± 1.3	+	0.0 ± 0.0	−	.001	*μ*_*T*_ > *μ*_*C*_
Kids	0	0.0 ± 0.0	−	0.0 ± 0.0	−	1.000	*μ*_*T*_ = *μ*_*C*_
Kids	3	4.5 ± 1.3	+	0.0 ± 0.0	−	.001	*μ*_*T*_ > *μ*_*C*_
Kids	7	3.8 ± 1.2	+	0.0 ± 0.0	−	.001	*μ*_*T*_ > *μ*_*C*_
Kids	14	2.9 ± 0.4	+	0.0 ± 0.0	−	.001	*μ*_*T*_ > *μ*_*C*_
Kids	21	2.7 ± 0.4	+	0.0 ± 0.0	−	.001	*μ*_*T*_ > *μ*_*C*_
Kids	28	2.5 ± 0.3	+	0.0 ± 0.0	−	.001	*μ*_*T*_ > *μ*_*C*_
Kids	42	2.4 ± 0.5	+	0.0 ± 0.0	−	.001	*μ*_*T*_ > *μ*_*C*_
Kids	56	2.3 ± 0.5	+	0.0 ± 0.0	−	.001	*μ*_*T*_ > *μ*_*C*_
Kids	70	2.0 ± 0.7	+	0.0 ± 0.0	−	.001	*μ*_*T*_ > *μ*_*C*_
Kids	100	1.8 ± 0.5	+	0.0 ± 0.0	−	.001	*μ*_*T*_ > *μ*_*C*_
Kids	120	0.8 ± 0.6	−	0.0 ± 0.0	−	.009	*μ*_*T*_ > *μ*_*C*_

Abbreviations: −, negative; +, positive; *C*, control group; *T*, treatment group; VN, virus neutralization; VNI, virus neutralization index; *μ*, mean.

Kids: As shown in Table 1 and Figure 2, before receiving colostrum, VNI was not significantly different between the control and treatment groups (*P* > .05). On days 3, 7, 14, 21, 28, 42, 56, 70, 100 and 120 after birth, VNI in the kids in the treatment group was significantly higher than that of the kids in the control group (*P* < .05).

VNI was positive in does after vaccine administration until delivery (Table 1, Figure 2). The average of the serum antibody titer in the kids was positive up to 100 days (Table 1, Figure 2). The number of seronegative goat kids in the treatment group on days 56, 70, 100 and 120 after birth was 2 (males), 4 (2 males and 2 females), 5 (3 males and 2 females), and 15 (8 males and 7 females), respectively.

## 4 DISCUSSION

In the present study, the vaccine resulted in positive serum VN ability in all vaccinated female goats. This positive serum VN ability was stable for 6 months (until parturition). After receiving sufficient colostrum, all goat kids born from vaccinated goats in the study had VN antibodies. The first kids with negative serum VN ability were observed on the 56th day after birth. On the 120th day after birth, all the goat kids had negative serum VN. The average serum VN antibody of the studied kids reached below the cut‐off at the age of 100 to 120 days after birth.

Seropositivity of goats against GTPV persists for 6 months after vaccination,^21^, ^22^ and the results of these studies are consistent with the present study. The results of the present study showed that the stability of antibody levels against GTP in does was acceptable from the time of vaccine administration until the parturition time (6 months). This shows that administration of GTP vaccine around the time of parturition is not required, and its annual administration is an appropriate measure. This phenomenon is helpful in health management of goat farms because during late pregnancy (2 months before delivery), the farm veterinarian focuses on prescribing other vaccines (such as enterotoxemia, contagious agalactia, etc.) while also managing other problems, such as hypocalcemia, pregnancy toxemia.

Cellular immunity is more important than humoral immunity in goat pox disease,^23^, ^24^ but in various experimental studies, it has been shown that passive immunity is able to prevent clinical disease after experimental challenges.^12^ Based on experimental studies,^12^, ^23^ the small ruminant pox viruses in the body of newborn lambs after the initial multiplication at the entry site and the draining regional lymph node, the small ruminant pox viruses spread throughout the body of newborn lambs via the blood where presence of maternal antibodies capable of neutralizing may prevent clinical disease. These studies demonstrate the importance of passive immunity in the newborn small ruminant in the prevention of small ruminant pox diseases.

In the present study, the antibody titer in newborns receiving sufficient colostrum was higher than the titer of does in late pregnancy. This phenomenon is reported^12^ and attributed to the active transfer of immunoglobulins from maternal blood to colostrum in late pregnancy.

The persistence of protective passive immunity in lambs born from vaccinated ewes is 60 days.^12^ Pregnant ewes were vaccinated 1 month before parturition, while in the present study, does were vaccinated 6 months before parturition. In this study, the used vaccine titration was 10^5.5^, while in the present study, the vaccine titration was 10^5.7^. In this study, a vaccine containing attenuated RM65 strain of sheep pox virus was used, while in the present study, a vaccine containing attenuated Gorgan strain of goat pox virus was used. In a study, it was concluded that 6 to 8 weeks of age is the appropriate age for vaccination against sheep pox in lambs born to vaccinated does.^25^

The term “window of susceptibility” is used to describe the period of time between the absence of passive immunity and the presence of vaccination‐induced immunity. The duration of this period is related to some factors including the breed, the amount of antibodies in the mother's colostrum and the amount of colostrum consumed and absorbed by neonates.^10^, ^26^ In this study, the susceptibility window of goat kids to GTP started as early as 56 days after birth in some kids and at most by 120 days after birth.

The present study shows that suitable time for the first vaccination against GTP in the goat kids receiving sufficient amount of colostrum from vaccinated does is 100 to 120 days after the birth. But this finding applies to industrial farms where there is strong monitoring of receiving sufficient colostrum.

## 5 CONCLUSIONS

Based on the findings and discussion, in the goat kids receiving adequate colostrum and born from vaccinated does, GTP vaccine administration around 100 to 120 days could be a reasonable window based on VNI. In these goat kids, the window of susceptibility to GTP starts at the age of 56 days and is completed at the age of 120 days.

## CONFLICT OF INTEREST DECLARATION

Authors declare no conflict of interest.

## OFF‐LABEL ANTIMICROBIAL DECLARATION

Authors declare no off‐label use of antimicrobials.

## INSTITUTIONAL ANIMAL CARE AND USE COMMITTEE (IACUC) OR OTHER APPROVAL DECLARATION

Ethics code in the research was taken from Ajdad Sepidan Kowsar company research committee (ethics code: 10219‐02/01/29).

## HUMAN ETHICS APPROVAL DECLARATION

Authors declare human ethics approval was not needed for this study.
